# Socioeconomic Inequities in Abdominal Aortic Aneurysm Screening Uptake Among Women: A Narrative Review

**DOI:** 10.7759/cureus.99655

**Published:** 2025-12-19

**Authors:** Abimbola O Kolawole, Fahd Mohamed, Denise Mourad

**Affiliations:** 1 Medicine, Central Michigan University College of Medicine, Mount Pleasant, USA; 2 Internal Medicine, Central Michigan University College of Medicine, Mount Pleasant, USA; 3 Internal Medicine, Central Michigan University College of Medicine, Saginaw, USA

**Keywords:** abdominal aortic aneurysm, cardiovascular disparities, screening inequity, socioeconomic factors, united states, women

## Abstract

Abdominal aortic aneurysm (AAA) is a life-threatening vascular condition with higher rupture risk and poorer surgical outcomes in women compared to men. U.S. screening programs predominantly target men, potentially contributing to underdiagnosis among high-risk women. Socioeconomic factors may influence disparities in AAA screening participation.

The aim of this narrative review is to synthesize evidence on socioeconomic determinants influencing AAA screening uptake among women in the United States and to explore potential equity-centered strategies.

A narrative review was conducted using PubMed, MEDLINE, Scopus, and Google Scholar for U.S.-based studies published between January 1990 and August 2025. Search terms included “abdominal aortic aneurysm,” “AAA screening,” “women,” “socioeconomic status,” “income,” “education,” “health disparities,” and “screening uptake.” Eligible studies included observational studies, cohort studies, retrospective analyses, policy reviews, and meta-analyses reporting AAA outcomes stratified by sex and at least one socioeconomic factor. Two reviewers independently screened studies and extracted data on study characteristics, AAA outcomes, and socioeconomic determinants. Findings were synthesized narratively.

Thirty-two studies met inclusion criteria. Evidence suggests that women experience lower AAA screening rates, influenced by guideline exclusions and intersecting socioeconomic factors. Lower income, limited education and health literacy, underinsurance, rural residence, and race/ethnicity were consistently associated with reduced screening uptake and delayed diagnosis. While the strength and consistency of associations varied across studies, socioeconomic determinants appeared to interact with female sex, potentially contributing to later-stage presentation and higher rupture risk.

Socioeconomic inequities are associated with disparities in AAA screening among women. Integrating socioeconomic factors into screening policy and clinical practice, alongside targeted interventions, may help improve equity in detection. Future research should prioritize sex- and socioeconomic status (SES)-disaggregated data and evaluate the effectiveness of interventions designed to enhance screening access for high-risk women.

## Introduction and background

Abdominal aortic aneurysm (AAA) is a progressive, life-threatening vascular disease characterized by permanent dilation of the abdominal aorta, typically defined as a diameter of 3.0 cm or greater [1]. Although historically considered a predominantly male condition, AAA in women carries a disproportionately higher risk of rupture and mortality at smaller aneurysm diameters compared to men [2]. Women also experience poorer surgical outcomes and are more frequently denied elective repair due to late presentation, smaller vessel anatomy, and higher perioperative risks [3]. These sex-specific differences, combined with limited baseline screening opportunities, underscore the critical importance of timely AAA detection in women.

Despite evidence of AAA prevalence in high-risk women, socioeconomic inequities continue to limit screening uptake. Organized ultrasound screening reduces aneurysm-related mortality in men, yet women have largely been excluded from major national screening trials and guideline recommendations [4,5]. Current U.S. screening policies, including the 2019 U.S. Preventive Services Task Force (USPSTF) guidelines, primarily recommend one-time AAA screening for men aged 65-75 years who have ever smoked, offering only selective screening for women with significant risk factors [5]. These male-focused criteria inherently restrict routine screening for women. Socioeconomic determinants - including low income, limited education and health literacy, underinsurance, rural residence, and race/ethnicity - interact with guideline exclusions and biological sex differences to further limit preventive care engagement, risk assessment, and access to vascular imaging [6,7]. Women experiencing multiple layers of disadvantage face compounded barriers, increasing the likelihood of delayed AAA detection, late-stage presentation, and adverse outcomes.

Despite growing recognition of AAA risk among high-risk women, no U.S.-focused review has systematically examined how intersecting social determinants shape screening inequities. We selected a narrative review approach because the literature is heterogeneous, encompassing observational studies, cohort analyses, and policy evaluations with diverse methodologies, populations, and outcome measures. Narrative synthesis allows for an integrated, intersectional exploration of socioeconomic and sex-based determinants while identifying patterns, gaps, and implications for equity-focused screening strategies.

This review evaluates the influence of income, education, insurance coverage, geographic access, race and ethnicity, smoking history, and healthcare utilization on AAA screening participation among women. By highlighting these gaps, the review aims to inform the development of risk-based, equity-centered strategies to improve detection and management of AAA in women.

## Review

Methods

Literature Search Strategy

A structured literature search was conducted for studies published from January 1990 to August 2025 in PubMed, MEDLINE, Scopus, and Google Scholar. Google Scholar was included to identify gray literature, with all records screened using consistent eligibility criteria. Both MeSH terms and free-text keywords were applied. Search terms included: “abdominal aortic aneurysm,” “AAA screening,” “women,” “sex differences,” “socioeconomic status,” “income,” “education,” “health disparities,” “screening uptake,” and “United States.”

Study Selection

The search retrieved 312 records. After removing duplicates, 284 records were screened by title and abstract. Full-text review was conducted for 54 articles, of which 32 studies were included in the final synthesis. Study selection was performed independently by two reviewers, and discrepancies were resolved through discussion.

Eligibility Criteria

Studies were eligible if they were U.S.-based and included observational studies, cohort studies, retrospective analyses, policy reviews, or meta-analyses reporting AAA outcomes stratified by sex and at least one socioeconomic factor. International studies were reviewed selectively for contextual comparison but were not included in the primary synthesis.

Data Extraction and Synthesis

Data extracted included study design, population characteristics, AAA outcomes, and socioeconomic determinants such as income, education, insurance status, geographic location, race/ethnicity, and health system engagement. Themes were identified iteratively using a narrative synthesis approach. Greater interpretive weight was given to studies with larger cohorts, robust methodology, and clearly defined outcomes. This approach allowed identification of consistent patterns of AAA screening disparities among women and informed recommendations for equity-centered screening strategies.

Results

Socioeconomic, Demographic, and Structural Determinants of AAA Screening in Women

Across the 32 studies included in the final synthesis, multiple determinants were associated with disparities in AAA screening among women. These determinants - household income, education, insurance status, geographic access, smoking prevalence, and race/ethnicity - frequently co-occur, complicating efforts to isolate independent effects. Most studies were observational, including cross-sectional analyses, retrospective cohort studies, and population-based registry analyses, which limits causal inference. Observed associations should therefore be interpreted as correlations rather than evidence of causation. Effect sizes varied substantially across studies, and several reported null or attenuated associations after adjustment for confounding variables.

Income, Education, and Health Literacy

Lower household income consistently correlated with reduced AAA screening participation [7-9]. Multiple studies reported that women in the lowest income quartiles were 25-45% less likely to receive screening compared with higher-income counterparts [7,9]. Cost-related barriers included direct costs for imaging follow-up, transportation, and lost wages from time off work. However, some studies noted that these associations attenuated when insurance status was accounted for, indicating partial mediation [9].

Educational attainment and health literacy were similarly associated with screening uptake [10-13]. Women with a high school education or less had lower awareness of AAA and fewer preventive care interactions, which reduced the likelihood of screening referrals [12,13]. Several studies highlighted that health literacy affected not only awareness but also comprehension of screening eligibility and interpretation of risk, particularly in settings where clinicians assumed baseline knowledge of vascular disease [14-16]. Contradictory findings were reported in a few regional studies where educational attainment did not independently predict screening uptake after adjusting for insurance and income, suggesting complex interplay among socioeconomic factors [11,13].

Health Insurance Status

Insurance coverage emerged as a major determinant of screening access [17-20]. Uninsured and underinsured women were consistently less likely to receive AAA screening, with some studies reporting odds ratios ranging from 0.4-0.6 compared with insured women [19,20]. Even among Medicare beneficiaries, women were substantially less likely than men to undergo screening, with disparities persisting after adjustment for age, comorbidities, and smoking history [18]. Effect sizes were heterogeneous across studies, with smaller regional cohorts showing more pronounced sex-based inequities compared to large national datasets.

Intersection with socioeconomic status (SES) was notable: women from low-income households were overrepresented among Medicaid beneficiaries or the uninsured, and screening uptake was lower in these groups [10,20]. Variability across state Medicaid programs further contributed to inconsistent coverage and uptake. Some studies reported null associations between insurance type and screening when controlling for geographic access, highlighting the overlapping influence of structural and socioeconomic factors [10,20].

Geographic and Structural Barriers

Geographic access to vascular care, including rural residence and proximity to imaging centers, was associated with lower screening uptake [9,21]. Women in rural areas were 30-50% less likely to receive AAA screening than urban counterparts in several studies [21]. These barriers often intersected with lower SES, reduced educational attainment, and limited transportation options. Some studies suggested that geographic effects were partially mediated by referral patterns and local healthcare infrastructure, while others reported persistent rural disparities even when accounting for SES and insurance [9,22].

Healthcare infrastructure shortages, including the limited availability of vascular specialists and on-site imaging, were frequently noted. Several studies emphasized that rural hospitals’ lack of specialty coverage contributed to delayed referrals and lower screening initiation, particularly among older women with mobility or caregiving constraints [10,18]. Null findings were reported in a small subset of studies where mobile screening units or telehealth interventions partially mitigated rural access barriers [9].

Smoking History and Eligibility Bias

Smoking is the strongest modifiable risk factor for AAA development and progression, increasing aneurysm risk up to fivefold through mechanisms of chronic vascular inflammation, elastin degradation, and medial wall weakening [23]. Smoking status is a key eligibility criterion under USPSTF AAA screening guidelines, yet reporting and documentation were inconsistent across studies [20,24-27]. Disadvantaged women were more likely to smoke, yet screening rates did not consistently reflect this elevated risk. Observed associations were inconsistent: some studies found a moderate positive association between smoking and screening when women were identified as eligible, while others reported no association, likely reflecting under-documentation of smoking history or gaps in provider referral practices [25].

The interaction of smoking with SES and geographic factors was notable. Low-income women who smoked were frequently underrepresented in screening cohorts, suggesting structural barriers and provider-level factors limited uptake despite elevated risk [10,26,27]. Contradictory findings across regions indicate that contextual factors, including local provider practices and population-level smoking prevalence, affect the relationship between tobacco exposure and screening participation.

Race, Ethnicity, and Intersectional Inequities

Racial and ethnic disparities were consistently reported, with Black, Hispanic, and Native American women less likely to receive AAA screening than White women [15,16,22,28-31]. Effect sizes varied, with some studies reporting a 40-60% reduction in screening odds among Black women. However, disentangling race from SES, insurance, and geographic access was rarely possible. In studies that adjusted for SES and insurance, racial disparities were often attenuated but not fully eliminated, indicating both overlapping and potentially independent effects [14,22].

Structural and systemic factors - including lower provider referral rates, underinsurance, geographic isolation, and cumulative social disadvantage - were frequently cited as contributors to inequity. Contradictory findings were observed in a few studies where race differences diminished after controlling for access and SES, highlighting challenges in separating individual and structural determinants [22].

Integrated Patterns Across Determinants

Across the included studies, evidence indicates that AAA screening disparities in women result from intersecting socioeconomic, demographic, and structural factors rather than any single determinant. Lower income, limited education, underinsurance, rural residence, smoking, and non-White race each showed associations with reduced screening, but effect sizes varied and were frequently attenuated when adjusted for overlapping determinants. Contradictory or null findings underscore the complexity of interpreting observational data and the importance of considering multilevel interactions. Collectively, these studies suggest that screening inequities are multifactorial, with compounded effects of socioeconomic disadvantage, structural barriers, and demographic factors contributing to lower AAA screening participation among women.

Discussion

Socioeconomic Interactions With Gender: Compounding Disparities in AAA Screening

Women in the United States face a dual burden in AAA detection: limited baseline screening eligibility under current guidelines and heightened socioeconomic vulnerability. Intersectional analysis demonstrates that socioeconomic determinants - including income, education, insurance status, and geographic access - interact to amplify sex-specific disparities in screening uptake and timely diagnosis [7,9]. Women with low income or underinsurance are less likely to attend preventive care visits, reducing opportunities for AAA risk assessment and referral. Lower education levels and limited health literacy further mediate awareness of AAA risk, comprehension of screening recommendations, and the ability to navigate complex healthcare systems [8,21].

Geographic barriers compound these inequities. Rural residence correlates with lower AAA screening rates due to reduced local availability of imaging services and specialist referrals [21]. When combined with female sex, these geographic disadvantages disproportionately affect older women, who are more likely to experience social isolation, transportation challenges, or caregiving responsibilities [9].

Race and ethnicity reflect structural socioeconomic disadvantage in the U.S. context. Women from historically marginalized communities are more likely to experience underinsurance, limited specialty care access, and systemic barriers within healthcare institutions, contributing to delayed AAA detection and higher rates of emergent surgical presentations compared to White women [7,32]. These disparities are magnified when intersecting with low income, rural residence, or low education.

Smoking, a primary AAA risk factor, interacts with SES and gender. Women from lower-income groups or with lower educational attainment have higher smoking prevalence and encounter additional barriers to cessation support, further increasing risk for AAA development and rupture [32].

Together, these factors suggest that AAA screening inequity arises from the complex interplay of sex, socioeconomic status, race, and geographic context, rather than any single determinant. Women experiencing multiple layers of disadvantage face compounded risk for missed screening, delayed diagnosis, and adverse outcomes. Table 1 outlines socioeconomic determinants linked to AAA screening in women.

**Table 1:** Summary of Socioeconomic Determinants Linked to Abdominal Aortic Aneurysm (AAA) Screening Inequity in Women SES: socioeconomic status

Socioeconomic Factor	Impact on Screening Uptake	Key References
Income / Financial barriers	Reduced preventive care access, missed appointments	Carter et al., 2020 [7]; Duncan et al., 2021 [9]
Education / Health literacy	Lower awareness of AAA risk, difficulty navigating healthcare	Patel et al., 2023 [8]; Summers et al., 2024 [21]
Insurance status	Underinsurance or Medicaid limits referral and follow-up	Kirby et al., 2021 [33]
Geographic location	Rural residence limits access to imaging and specialists	Sherry et al., 2021 [10]; Duncan et al., 2021 [9]
Race/Ethnicity (SES proxy)	Higher structural barriers to care, delayed detection	Carter et al., 2020 [7]; Welsh et al., 2025 [32]
Smoking prevalence	SES-linked higher smoking rates in women, increased AAA risk	Welsh et al., 2025 [32]

Policy Implications and Recommendations

Current AAA screening guidelines in the United States primarily target men aged 65-75 years with a history of smoking, offering only selective recommendations for women with significant risk factors [5]. This male-focused paradigm contributes to delayed detection and higher rupture-related mortality among women. Incorporating sex-specific risk factors - such as faster aneurysm growth rates, smaller baseline aortic diameters, and increased rupture susceptibility - into eligibility criteria may improve early detection and outcomes [2,3].

Socioeconomic disparities further exacerbate inequities. Policy interventions should prioritize access for low-income, underinsured, and rural women. Potential strategies include expanding Medicare and Medicaid coverage beyond current male-focused recommendations, reducing cost-sharing for high-risk women, and funding mobile screening units targeting underserved regions [9,21]. To support outreach and improve screening access for socioeconomically disadvantaged women, practical tools such as Social Vulnerability Indices (SVIs) can identify high-risk communities, and community health worker programs can facilitate education, navigation of healthcare services, and referral to screening.

Outreach and education campaigns aimed at improving health literacy and awareness of AAA risk among women could further increase screening uptake. Clinicians should integrate AAA risk assessment into routine preventive visits for women with smoking history or other risk factors, with focused attention on socioeconomically disadvantaged populations. Telehealth-based risk stratification and automated referral reminders may help mitigate geographic and transportation barriers, particularly in rural or resource-limited settings.

Future guidelines should adopt intersectional frameworks that consider both sex and socioeconomic determinants. Equity-centered approaches may include risk-scoring systems that weight socioeconomic disadvantage alongside traditional AAA risk factors, ensuring high-risk women are identified and referred regardless of baseline prevalence differences. Research and policy development should continue to prioritize the collection of sex- and SES-disaggregated data to evaluate intervention effectiveness, monitor implementation success, and track reductions in AAA-related disparities.

Proposed Screening Eligibility Expansion for High-Risk Women

Expanding AAA screening eligibility to incorporate both sex-specific risk and socioeconomic disadvantage may reduce disparities in timely detection. Table 2 outlines proposed criteria based on U.S.-based epidemiologic evidence, including age, smoking history, family history, SES, comorbidities, and health system engagement [2,3,7,9,11,32,34]. This multidimensional framework emphasizes biological, behavioral, and social determinants to support equity-centered screening programs, mobile imaging units, and targeted outreach for underserved populations.

**Table 2:** Proposed Abdominal Aortic Aneurysm (AAA) Screening Eligibility Expansion for High-Risk Women COPD: Chronic Obstructive Pulmonary Disease, SES: Socioeconomic Status

Risk Factor	Current USPSTF Criteria	Proposed Expansion for Women	Rationale
Age	65–75 years (men)	60–75 years (women with risk factors)	Earlier detection due to accelerated growth and higher rupture risk in women (Lancaster et al., 2022) [3]
Smoking History	Ever smoked (men)	Ever smoked or current smokers (women)	Smoking remains strongest modifiable risk factor; SES interacts with smoking prevalence (Welsh et al., 2025) [32]
Family History	First-degree relative with AAA (men)	First-degree relative with AAA (women)	Genetic predisposition applies equally to women; early referral warranted (Adams et al., 1993) [34]
Socioeconomic Status	Not included	Low income, underinsured, or rural residence	SES barriers contribute to reduced preventive care engagement and delayed diagnosis (Carter et al., 2020; Duncan et al., 2021) [7,9]
Comorbidities	Not included	Hypertension, COPD, or cardiovascular disease	Comorbidities increase AAA risk and mortality; targeted screening may reduce adverse outcomes (Ramkumar et al., 2020) [2]
Health System Engagement	Not included	Limited primary care utilization	Women with infrequent preventive visits may benefit from direct screening outreach (Bailey et al., 2017) [11]

Strengths and limitations

This review synthesizes evidence from 32 studies spanning diverse U.S. populations, providing an intersectional perspective on socioeconomic determinants of AAA screening in women. Strengths include the integration of multiple risk factors, explicit acknowledgment of overlapping disparities, and the critical evaluation of study designs, effect sizes, and consistency across findings.

Limitations include reliance on observational studies, which preclude causal inference, and heterogeneity in study populations, methodologies, and reporting of SES measures. Several studies reported null or contradictory findings that highlight uncertainty in effect magnitude. Additionally, data on non-White women are limited, and few studies disaggregate outcomes by both sex and socioeconomic status, potentially underestimating compounded disparities. Despite these limitations, the review provides a comprehensive foundation for equity-focused research, policy, and clinical guidance.

Future research

Key gaps remain in understanding and addressing socioeconomic disparities in AAA screening among women. Prospective studies and intervention trials are needed to evaluate strategies that improve screening uptake in socioeconomically disadvantaged populations. Research should prioritize sex- and SES-disaggregated data across diverse racial, ethnic, and geographic groups to clarify independent and intersecting effects. Studies exploring mechanisms - such as provider referral patterns, healthcare utilization, and structural barriers - could inform targeted interventions. Evaluations of mobile screening, telehealth, and educational campaigns are needed to assess both uptake and AAA-related outcomes. Finally, developing intersectional risk models integrating sex, socioeconomic factors, and comorbidities may guide equity-centered screening policies.

## Conclusions

Socioeconomic factors are associated with disparities in AAA screening among women in the United States. Observational evidence suggests that women with lower income, limited education, underinsurance, or geographic barriers are less likely to receive screening and may experience delayed diagnosis. While current guidelines are largely based on male risk patterns, the magnitude and consistency of sex- and SES-related effects on AAA outcomes vary across studies. These findings highlight the need for further research, sex- and SES-disaggregated reporting, and consideration of socioeconomic determinants in screening policy and risk assessment. Targeted interventions may help reduce inequities, though evidence on their effectiveness remains limited.
